# Interactions of Anaerobic Bacteria with Dental Stem Cells: An *In Vitro* Study

**DOI:** 10.1371/journal.pone.0110616

**Published:** 2014-11-04

**Authors:** Anne Biedermann, Katja Kriebel, Bernd Kreikemeyer, Hermann Lang

**Affiliations:** 1 Department of Operative Dentistry and Periodontology, University of Rostock, Rostock, Germany; 2 Institute of Med. Microbiology, Virology and Hygiene, University of Rostock, Rostock, Germany; Boston University, United States of America

## Abstract

**Background:**

In patients with periodontitis, it is highly likely that local (progenitor) cells encounter pathogenic bacteria. The purpose of this *in vitro* study was to elucidate how human dental follicle stem cells (hDFSC) react towards a direct challenge with anaerobic periodontal pathogens under their natural oxygen-free atmosphere. HDFSC were compared to human bone marrow mesenchymal stem cells (hBMSC) and differentiated primary human gingival fibroblasts (hGiF), as well as permanent gingival carcinoma cells (Ca9-22).

**Methodology/Principal Findings:**

The different cell types were investigated in a co-culture system with *Porphyromonas gingivalis (P. gingivalis)* and *Fusobacterium nucleatum (F. nucleatum)*. The viability of the cells and pathogens under anaerobic conditions, as well as interactions in terms of adherence and internalization, were examined. Additionally, the release of pro-inflammatory interleukin-8 (IL-8) and anti-inflammatory interleukin-10 (IL-10) was quantified via enzyme-linked immunosorbent assay. The bacteria adhered less efficiently to hDFSC compared to Ca9-22 (*P. gingivalis*: 0.18% adherence to hDFSC; 3.1% adherence to Ca9-22). Similar results were observed for host cell internalization (*F. nucleatum*: 0.002% internalization into hDFSC; 0.09% internalization into Ca9-22). Statistically significantly less IL-8 was secreted from hDFSC after stimulation with *F. nucleatum* and *P. gingivalis* in comparison with hGiF (*F. nucleatum*: 2080.0 pg/ml – hGiF; 19.7 pg/ml – hDFSC). The IL-10 response of the differentiated cells was found to be low in relation to their pro-inflammatory IL-8 response.

**Conclusions/Significance:**

The results indicate that dental stem cells are less prone to interactions with pathogenic bacteria than differentiated cells in an anaerobic environment. Moreover, during bacterial challenge, the stem cell immune response seems to be more towards an anti-inflammatory reaction. For a potential future therapeutic use of hDFSC, these findings support the idea of a save application.

## Introduction

To determine the biological role of dental stem cells in their natural environment and to examine the save potential therapeutic application for tissue regeneration, the understanding of cell–bacteria interactions is of great importance [1].

The periodontal tissues are composed of a variety of different cell types. Recently, stem cells have been reported to be part of this cellular environment [2], [3]. Generally, stem cells have two major characteristics that distinguish them from other cells: they are capable of self-renewal, and upon division, they give rise to cells that have the potential to differentiate [4].

The dental follicle harbors human dental follicle stem cells (hDFSC), and is present in impacted teeth, which are commonly extracted and disposed of medical waste in dental practice [5]. Therefore, these cells are an easily accessible cell source for experimental and future clinical applications in periodontal tissue or bone regeneration approaches [6], [5].

The periodontal pocket is predisposed towards interdependencies of local cells and putative pathogens, which have been one focus of periodontal research [7]. More than 700 different bacterial species can be found in the oral cavity [8]. The majority settles in multiple different forms of biofilm patterns. Once detached from the biofilm, some of the bacteria penetrate into deeper tissues and may disturb host cell homeostasis [7]. Direct contact between bacteria and human cells triggers the expression of a variety of immune response mediators [9], [7]. Interleukin (IL)-8 is a pro-inflammatory cytokine and is predominantly secreted by epithelial cells. It has been suggested to be involved in the local host defense mechanisms leading to neutrophil migration [9]. This chemoattractant activates and attracts neutrophils to the site of infection or injury [10], [11], [9]. As a regulatory mechanism, the expression of the anti-inflammatory IL-10 terminates the secretion of pro-inflammatory cytokines and reduces the expression of major histocompatibility complex class II and co-stimulatory molecules [12]. The critical role of IL-10 in infectious disease appears to be the modulation of inflammatory responses to microbial pathogens [13].

The pathogenic bacteria *F. nucleatum* and *P. gingivalis* can frequently be found in diseased periodontal tissues and they play important roles in the initiation and progression of periodontitis [14], [15]. *P. gingivalis* is able to induce strong cytokine and chemokine expression in gingival epithelial and other host cells, which has been shown to positively correlate with the adhesive/invasive potential of the infecting strain [16]. *F. nucleatum* and *P. gingivalis* are obligate anaerobic bacteria that can only exist in areas of the oral cavity with low oxygen partial pressure (*e.g.*, the dental pocket). However, functional interaction studies of hDFSC and oral pathogenic bacteria in their natural anaerobic atmosphere are lacking.

Thus, the focus of our study was to investigate the interactions of hDFSC with periodontal pathogens. The results were compared with cells at various stages of differentiation: human bone marrow mesenchymal stem cells (hBMSC), human gingival fibroblasts (hGiF), and the permanent gingival carcinoma cell line Ca9-22. We hypothesized that hDFSC might show a different immunological response than differentiated cells after co-culture with periodontal pathogens because of their anti-inflammatory and tissue regenerative function in the injured or inflamed tissue [17]. Stem cells have to remain operative even in sites of chronic inflammation [1], and therefore, they might also be more tolerant towards bacterial stimuli.

## Material and Methods

### Cell Isolation and Culture

The stem cells were obtained from donors undergoing surgical treatment at either the Department of Cardiac Surgery or the Department of Oral Surgery at the University of Rostock. This study conforms to the Declaration of Helsinki, and all cell donors gave their informed written consent. The experimental protocol and further experiments were reviewed and approved by the Ethics Committee of the University of Rostock (No. A 2011 119 and No. A 2011 91).

Ca9-22 was provided by the Japanese Collection of Research Bioresources Cell Bank Osaka, Japan. The cells were cultured in DMEM supplemented with 10% FCS in cell culture flasks (Greiner Bio-one, Frickenhausen, Germany) (5% CO_2_, 37°C).

HGiF were cultured in DMEM supplemented with 10% FSC (10% CO_2_, 37°C). Isolation and culturing of hBMSC was performed as described by Gaebel *et al.* [18], and hDFSC as described by Haddouti *et al.* [19]. The authenticity of the stem cells was confirmed by plastic adherence, and flow cytometric analysis (monoclonal antibodies against CD29, CD44, CD45, CD73 and CD90 using a FACS scan flow cytometer LSRII with CellQuest-Software, Becton Dickinson). Multipotency of the cells was shown through induction into osteoblasts, chondroblasts, and adipocytes as previously described [20].

### Bacteria

*P. gingivalis* W50 and W83 (DSMZ, Braunschweig, Germany), *F. nucleatum* ATCC 23726, and ATCC 25586 (American Type Culture Collection, Manassas, USA) were purchased from commercial providers. The bacteria were grown in PYG medium supplemented with 5 µg/ml hemin and 1% vitamin K in an anaerobic atmosphere (10% CO_2_, 10% H_2_, 80% N_2_).

### Anaerobic Co-Culture

Bacteria were tested for their survival in cell culture medium prior to the co-culture experiments. They were grown in PYG medium supplemented with 5 µg/ml hemin and 1% vitamin K to the stationary phase. Subsequently, bacteria were centrifuged, washed in PBS, and each bacterial suspension was diluted in DMEM 1∶10. The optical density (OD) was measured at 600 nm from timepoint 0 every hour to timepoint 12 h with a final measurement after 24 h.

Cells were tested for their tolerance towards oxygen-free conditions. Cell-cultures at the 4^th^ to 6^th^ passage were harvested and seeded at a density of 8×10^3^ cells/well in a 24-well culture plate, and maintained in 2 ml of medium. Subsequently, cells were grown in an anaerobic workstation (miniMACS, DWS Meintrupp, Lahden-Holte, Germany) at 37°C for 24, 48, and 76 h, and numbers of viable cells were counted in a Neubauer hemacytometer after trypan blue staining.

For co-culture, hDFSC, hBMSC, hGiF, and Ca9-22 were seeded at a density of 8×10^3^–1×10^4^ cells/well in a 24-well culture plate, and maintained in 1 ml medium. For each experiment, the final concentration of the bacterial suspension was determined by measurement of the optical density at 600 nm (OD_600_) to obtain 1×10^8^ cells/ml, and dilutions were made to achieve the desired MOI. The bacterial inoculum was confirmed by counting of the colony-forming units (CFU). After reaching confluence, the cells were infected with live bacteria with a multiplicity of infection (MOI) of 1∶100 and incubated at 37°C in an anaerobic atmosphere.

For fluorescence microscopy (BX60 microscope, Olympus, Hamburg, Germany) the samples were stained with BacLight Live/Dead (Molecular Probes, Eugene, USA). The staining was documented with an attached digital camera (Leica, Solms, Germany).

### Adherence and Internalization

The eukaryotic cells (8×10^3^ cells/ml) were cultured in DMEM and grown to a monolayer. The bacteria were grown in PYG medium at 37°C under an anaerobic atmosphere. The bacterial density was adjusted to MOI 1∶100 in DMEM and added to the cell monolayer. After 2 h, the cells were washed and subsequently detached by adding 200 µl 0.25% trypsin/0.5 mM EDTA for 5 min. To quantify bound bacteria, the cells were lysed with distilled water and the number of bacteria in the lysate was assessed by viable counts. For quantification of internalized bacteria, the monolayers were washed with PBS. Medium containing gentamicin (300 mg/ml) and metronidazole (200 mg/ml) was added after 2 h and incubated for another 2 h. After 4 h, the same procedure was performed to quantify the internalized bacteria. The number of viable bacteria was confirmed by counting CFUs on agar plates incubated under an anaerobic atmosphere at 37°C.

For scanning electron microscopy (SEM), coverslips with hDFSC co-cultures were fixed in a 2.5% glutardialdehyde solution. They were subsequently washed with 0.1 M sodium acetate buffer (pH 7.3) and dehydrated in a graded series of ethanol. Finally, coverslips were subjected to critical-point drying with CO_2_ (Critical Point Dryer, Emitech, Ashford, UK), sputter-coated with gold, and examined with an electron microscope (Zeiss DSM 960A, Jena, Germany).

### Cytokine Secretion

IL-8 and IL-10 concentrations were measured in the supernatant of the bacteria–cell co-culture after 1, 2, 4, and 24 h of incubation by enzyme-linked immunosorbent assay (ELISA), using a commercially available kit (BD OptEIA, BD Biosciences, San Diego, USA) according to the manufacturer's instructions.

### Statistical Analysis

All results are presented as means ± standard deviation (SD). Statistical analyses were carried out by T-test (Prism 6 for Windows, Version 6.01, GraphPad Software, Inc., San Diego, CA, USA). Differences were considered statistically significant at *p<0.05, **p<0.01. All experiments were performed with at least four biological replicates (n = 4).

## Results

### Anaerobic Co-Culture

**Figure 1:**
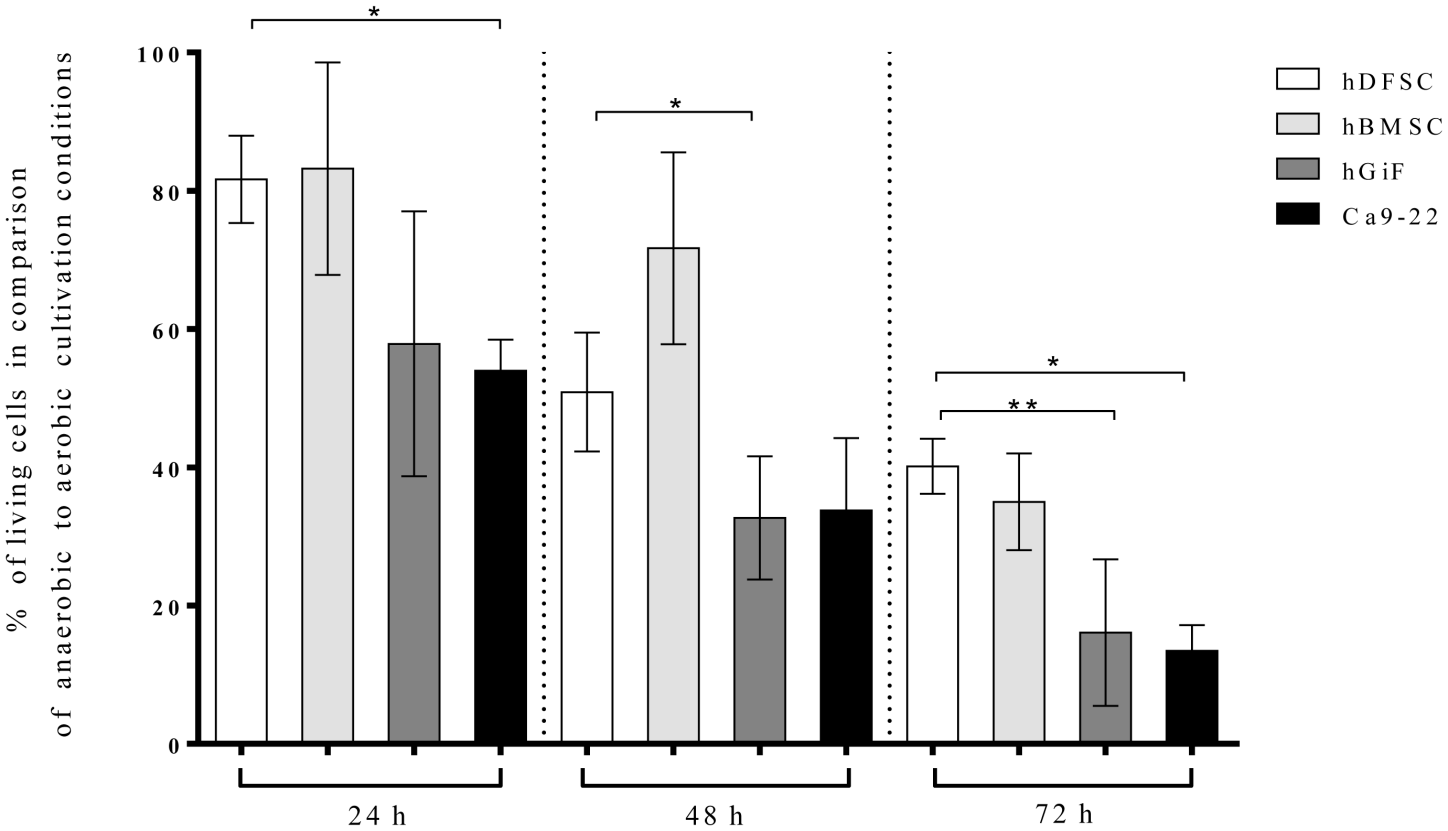
Cell survival under anaerobic conditions. Survival of hDFSC, hBMSC, hGiF and Ca9-22 cells under anaerobic compared with aerobic conditions. Anaerobic atmosphere: (10% CO_2_, 10% H_2_, 80% N_2_) and 37°C. Viable cells were counted at timepoints of 24, 48 and 72 h in a Neubauer hemacytometer by exclusion of trypan blue. Values are expressed as means ± SD (standard deviation), *p<0.05 (T-test), n = 4. Asterisks indicate statistically significant differences between hDFSC and hBMSC, hGiF, and Ca9-22.

The establishment of the anaerobic co-culture system as described by Kriebel *et al*. [21] included the verification of the cell survival under an anaerobic atmosphere prior to a bacterial challenge. Survival of the cells was evaluated after 12, 24 and 72 h (Fig. 1). Compared to aerobic conditions, 81.7% of the hDFSC, 83.2% of the hBMSC, 54.0% of the Ca9-22 cells, and 58.9% of the hGiF remained viable 24 h of an oxygen-free incubation. After 2 days, the hDFSC still showed higher survival rates than the Ca9-22: only 33.8% of Ca9-22 cells survived the 48-h incubation, whereas 50.9% of hDFSC cells were alive. The cell numbers decreased significantly for all cells after 72 h: hDFSC showed a survival of 40.2%, hBMSC 35.0%, hGiF 16.1%, and Ca9-22 13.5% (Fig. 1).

**Figure 2:**
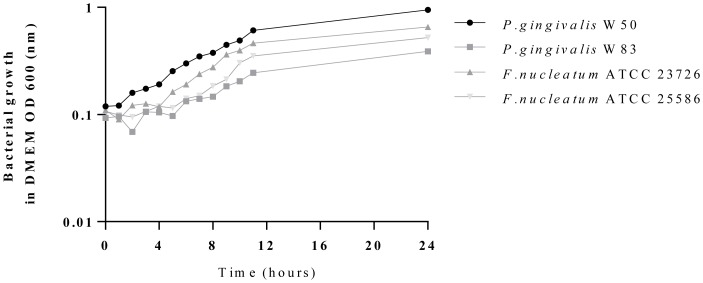
Bacterial growth in cell culture medium. Growth rates of bacteria in DMEM cell culture medium under anaerobic conditions (10% CO_2_, 10% H_2_, 80% N_2_), 37°C. Bacteria were grown in PYG medium supplemented with 5 µg/ml hemin and 1% vitamin K to the stationary phase. Subsequently, they were centrifuged, washed in PBS, and each bacterial suspension was diluted in DMEM 1∶10. The OD_600_ was measured at 600 nm from timepoint 0 every hour to timepoint 12 h with a final measurement after 24 h.

The bacterial growth was determined in cell culture medium under anaerobic conditions. All four tested species showed normal bacterial growth (Fig. 2). From these results, we concluded that co-culture with anaerobic bacteria and hDFSC (and hBMSC, hGiF, or Ca9-22) was possible. Live/dead staining and fluorescence microscopy confirmed the survival of bacteria and hDFSC in the co-culture system (data not shown).

### Adherence and Internalization

The human cells were separately challenged with live *P. gingivalis* W50, W83, *F. nucleatum* ATTC 23726, or ATTC 25586, employing a MOI of 1∶100. Attachment to and invasion of hDFSC by oral pathogenic bacteria was measured, and was compared with the results obtained with hBMSC, hGiF, and Ca9-22. All bacterial species were able to adhere to and invade the hDFSC. Notably, in all co-cultures, Ca9-22 cells showed the highest susceptibility to bacterial adhesion, which was statistically significant for *F. nucleatum* ATCC 2326 and *P. gingivalis* W50. Roughly the same number of bacteria adhered to the fibroblasts compared to the hDFSC. In contrast, in all co-cultures, the hBMSC were statistically significantly less prone to bacterial adhesion. SEM confirmed bacterial attachment to the hDFSC (Fig. S1 and S2).

**Figure 3:**
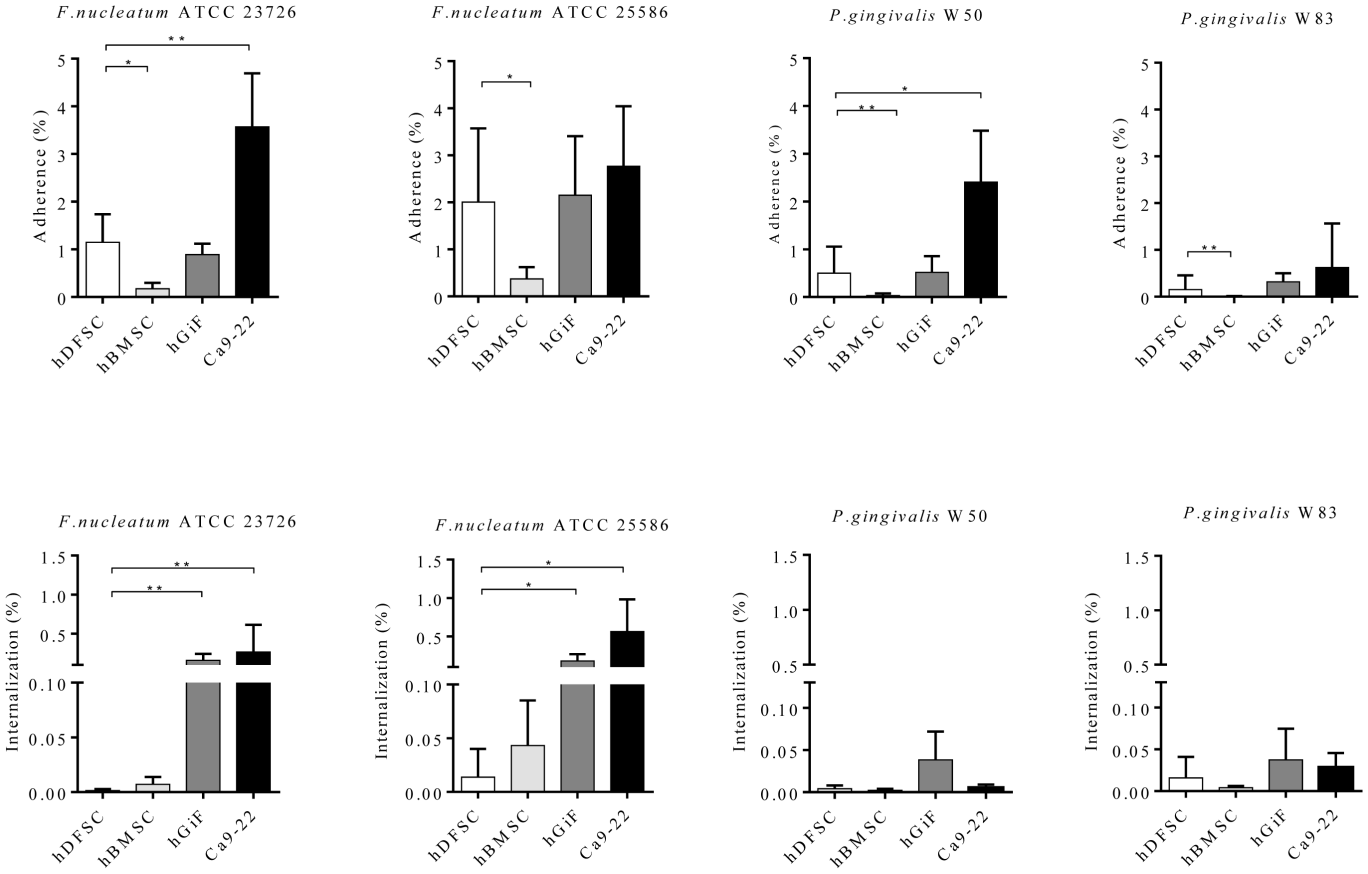
Adherence and internalization. The attachment levels are expressed as the percentage of live bacteria retrieved following cell lysis relative to the total number of bacteria at each timepoint. The assays were carried out as described in the text. Attachment levels of hDFSC were compared to hBMSC, hGiF, and Ca9-22. Values represent the means ± SD, *p<0.05, **p = 0.01 (T-test), n = 4.

The efficiency of the periodontal pathogens to internalize into the hDFSC was very weak. In hGiF and Ca9-22 co-cultures with *F. nucleatum* ATCC 23726 and *F. nucleatum* ATCC 25586, statistically significantly higher bacterial invasion rates were found compared with hDFSC. Both *P. gingivalis* strains appeared to be hardly invasive in all four cell lines (Fig. 3 and Table S1).

### IL-8 and IL-10 Secretion

**Figure 4:**
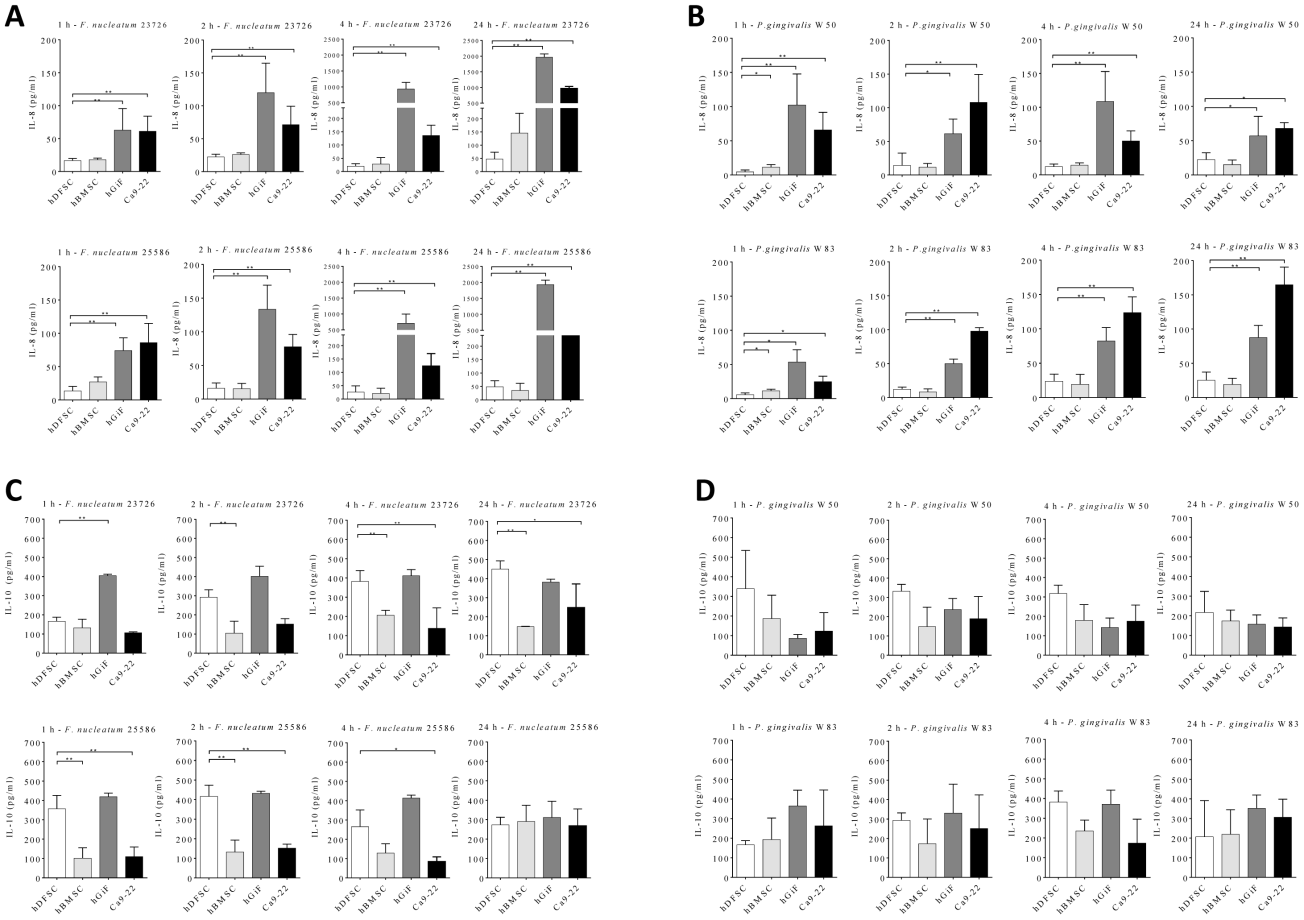
IL-8 and IL-10 secretion. The assays were carried out as described in the text. IL-8 and IL-10 levels were assayed by ELISA. Absorbance was read at 450 nm. Values represent the means ± SD, *p<0.05, **p = 0.01 (T-test), n = 4. Asterisks indicate statistically significant differences between hDFSC and hBMSC, hGiF, and Ca9-22. Cytokine secretion by hDFSC was compared with secretion by hBMSC, hGiF, and Ca9-22 cells at the same timepoint. **A**) IL-8 measured in the supernatant of the *F.nucleatum* ATCC 23727 and 25586 co-culture after 1, 2, 4, and 24 h. **B**) IL-8 measured in the supernatant of the *P. gingivalis* W50 and W83 co-culture after 1, 2, 4, and 24 h. **C**) IL-10 measured in the supernatant of the *F.nucleatum* ATCC 23727 and 25586 co-culture after 1, 2, 4, and 24 h. **D**) IL-10 measured in the supernatant of the *P. gingivalis* W50 and W83 co-culture after 1, 2, 4, and 24 h.

After co-culture with either live *F. nucleatum* or *P. gingivalis* (MOI 1∶100) for 1, 2, 4, and 24 h, IL-8 and IL-10 was detected in the supernatant of all four cell lines by ELISA (Fig. 4 and Tables S2 and S3). Unchallenged cells were used as a negative control.

Of note, there were statistically significantly lower IL-8 secretion levels in hDFSC and hBMSC supernatants compared with hGiF and Ca9-22. At all timepoints, hGiF consistently showed the highest IL-8 response (with the exception of at 1 h with *F. nucleatum* ATCC 255886 infection). Overall, *F. nucleatum* was the most efficient stimulatory bacterium for the secondary cytokine response. The strongest IL-8 response was noted after 24 h for hGiF co-cultured with *F.nucleatum* ATCC 23726, reaching 1964 pg/ml.

The initial IL-10 response (1 h) was higher than the initial IL-8 response measured for all cells. After 4 h of co-culture, the IL-8 response from hGiF and Ca9-22 increased significantly, whereas the hDFSC and hBMSC remained at almost the same level of IL-10 secretion, but showed only a small increase in IL-8 response.

## Discussion

The purpose of the present study was to comparatively characterize interactions between dental stem cell and periodontal pathogens *in vitro*, since in bacterially infected periodontal tissues, a mutual influence of local (progenitor) cells and anaerobic bacteria is highly likely [7]. This, to the best of our knowledge, has not yet been explored in a direct co-culture of hDFSC and periodontal pathogens. A typical challenge described for *in vitro* co-culture experiments with cells and anaerobic bacteria is the limited lifespan of these microorganisms under an aerobic atmosphere [22]. Hence, a co-culture system in which both the cells and anaerobic periodontal pathogenic bacteria were able to survive for a certain time-period was applied and selected cell–bacterial interactions were analyzed.

Host cell invasion is regarded as an important tool of pathogenic bacteria to afford protection from the host immune system, and this invasion contributes to tissue damage [23]. In fact, invasive bacteria have evolved a variety of mechanisms for host cell entry. Initially, the bacteria attach to the host cell membrane, followed by the induction of a series of structural and biochemical changes that facilitate bacterial penetration. Lamont *et al.* [23] demonstrated the invasion of *P. gingivalis* into gingival epithelial cells. It is known that *P. gingivalis* can recognize different host cell types and is capable of targeting different specific and distinct eukaryotic signaling pathways to induce uptake into the cell [24]. Additionally, the study of Han *et al.* [7] showed that *F. nucleatum* is able to adhere to and invade gingival epithelial cells.

In this study, we reveal the differential susceptibility of dental stem cells compared with hBMSC, hGiF, and Ca9-22 towards the very important bacterial pathogenicity traits of host cell adherence and internalization. A significantly lower bacterial adherence and internalization capacity was observed in dental stem cells compared with hGiF and the epithelial cell line Ca9-22. Variations in the cell-surface structure, as well as differences in the process of invasion, depending on the cell line and its level of differentiation might explain the decreased bacterial attachment and internalization found in hDFSC and hBMSC compared with hGiF or Ca9-22 co-cultures. Furthermore, the expression of varying amounts of specific cell-surface receptors that allow the attachment of pathogens may contribute to the different results for the adherence. Huard-Delcourt *et al*. [25] showed, via FACS-analysis, a different expression of receptors for *P. gingivalis* attachment for the two tested cell lines in that study (KB and gingival epithelial cells). It is not clear what kind of receptors were responsible, but these authors were able to show a saturation plateau for each cell line when all bacteria were bound. Whether PRRs (pathogen recognition receptors) play a role in this context still needs to be determined.

Moreover, a novel stem cell property has been described, which possibly contributes to a reduced bacterial adherence and internalization. BMSCs have recently been shown to impede *in vitro* pathogen growth [1]. Krasnodembskaya *et al.* [26] and Meisel *et al.* [27] described the production of the antibacterial peptide LL-37 by stem cells. If hDFSC also secrete LL-37 remains unclear.

To define differences in the immunological reactions of the cells after co-culture with *F. nucleatum* and *P. gingivalis*, the cytokine release was assessed. IL-8, an important signaling molecule in periodontitis [11], was chosen as being representative for the pro-inflammatory cytokine response. *In vitro*, IL-8 secretion is triggered by invading bacteria, particularly *F. nucleatum*; similar observations for *P. gingivalis* have been made, with different cell lines [28], [7]. Sandros *et al.* [16] showed that bacterial binding to the surface of epithelial cells leads to an increased expression of IL-8.

Our results show that after the co-culture of *P. gingivalis* and *F. nucleatum* with hDFSC, the IL-8 secretion was significantly less stimulated compared with hGiF and Ca9-22 cells. All four cell lines under investigation showed the highest IL-8 production after co-culture with *F. nucleatum* ATCC 23726. The decrease in IL-8 levels after 24 h in the *P. gingivalis* W50 co-culture is most likely due to the activity of cytokine degrading enzymes of this species [29]. An anti-inflammatory reaction was analyzed via measurement of the cellular IL-10 response. Initially, all cell types showed a higher IL-10 than IL-8 response. Of note, after 4 h of co-culture, the differentiated cells yielded a significantly higher IL-8 than IL-10 response, whereas the stem cells kept almost the same level of IL-10 secretion, but showed only a small increase in the IL-8 response. This means that hDFSC and hBMSC had higher IL-10 than IL-8 secretion at all measured timepoints.

The differences in the cytokine response between the cells might be rooted in the specificity of the innate immune system that is based on a broad spectrum of PRRs that recognize highly conserved pathogen-associated molecular patterns (PAMPs) [30], including Toll-like receptors (TLRs), the nucleotide-binding oligomerization domain proteins (Nods), and the G-protein-coupled receptors (GPCRs) [28]. By stimulating these receptors, the mitogen-activated protein kinase (MAPK) signaling pathway is activated leading to NF-κB nuclear translocation and resulting in cytokine expression [31]. TLR4 is activated by the LPS of Gram-negative species such as *F. nucleatum*, while *P. gingivalis* LPS signals via TLR2 [28]. Moreover, the fimbriae of *P.gingivalis* and gingipains also activate TLR4 [32]–[34]. The combined effect of the aforementioned activations and interactions could be responsible, at least in part, for the differences in cell response to various oral bacteria [28].

However, recent reports suggest that human dental mesenchymal stem cells, as well as hBMSC, possess immuno-regulating and anti-inflammatory properties [35], [36]. The *in vitro* findings of this work underline the potential immuno-modulating character of local dental progenitor cells in the context of bacterially induced inflammatory processes.

## Conclusion

We demonstrate that hDFSC (and hBMSC) show less interaction with periodontal pathogens concerning adherence and internalization. Moreover, in this experimental setup, the cytokine secretion of these cells favored an anti-inflammatory reaction. *In vitro*, we compared hDFSC, hBMSC, differentiated primary gingival fibroblasts, and the permanent tumor cell line Ca9-22 in an anaerobic environment. The results of this study indicate that there are differences between cell lines at various stages of differentiation concerning the tolerance towards bacterial infections. For future potential dental stem cell therapies, the findings presented here might be of clinical relevance.

However, better anaerobic survival of the stem cells could lead to a better chance of tissue regeneration after infection and damage, which seems to be reasonable from an evolutionary perspective. With respect to dental tissues, it can be concluded that hDFSC show a similar behavior to hBMSC in this study.

## Supporting Information

Figure S1 **Scanning electron microscope images of** ***F. nucleatum*** **ATCC 23727 attachment to hDFSC.**
(TIF)

Figure S2 **Scanning electron microscope images of** ***F. nucleatum*** **ATCC 23727 attachment to hDFSC.**
(TIF)

Table S1 **Adherence and internalization.** The attachment and invasion levels are expressed as the percentage of live bacteria retrieved following cell lysis relative to the total number of bacteria at each timepoint. The assays were carried out as described in the text. Attachment and invasion levels of hDFSC and hBMSC were compared with Ca9-22 cells. Values represent the means ± SD, *p<0.05, **p = 0.01 (T-test), n = 4.
(XLSX)

Table S2 **IL-8 secretion.** IL-8 levels were assayed by ELISA. Absorbance was read at 450 nm. Values represent the means ± SD, *p<0.05, **p = 0.01 (T-test), n = 4.
(XLSX)

Table S3 **Il-10 secretion.** IL-8 levels were assayed by ELISA. Absorbance was read at 450 nm. Values represent the means ± SD, *p<0.05, **p = 0.01 (T-test), n = 4.
(XLSX)
