# Supplementary figures and images for: Interactions of Anaerobic Bacteria with Dental Stem Cells: An In Vitro Study
Source: PLoS One. 2014 Nov 4;9(11):e110616. doi: 10.1371/journal.pone.0110616 (PMC4219685; doi:10.1371/journal.pone.0110616)

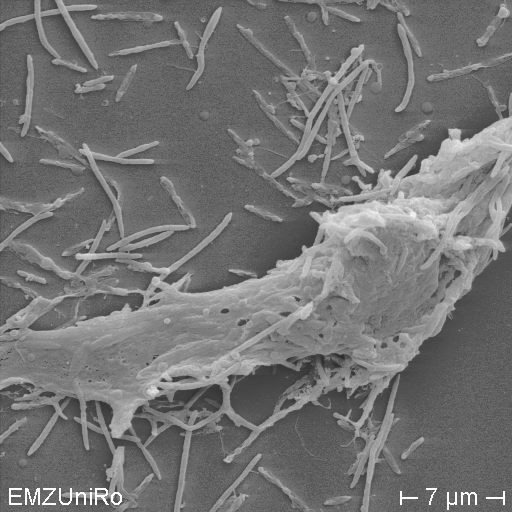

Supplement: Figure S1 — Scanning electron microscope images of F. nucleatum ATCC 23727 attachment to hDFSC. (TIF) [file pone.0110616.s001.tif]

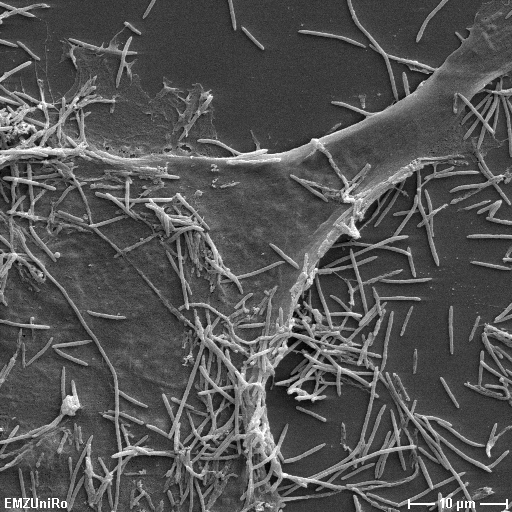

Supplement: Figure S2 — Scanning electron microscope images of F. nucleatum ATCC 23727 attachment to hDFSC. (TIF) [file pone.0110616.s002.tif]
